# Supplementary material for: Drug Shelf Life and Release Limits Estimation Based on Manufacturing Process Capability
Source: Pharmaceutics. 2023 Mar 26;15(4):1070. doi: 10.3390/pharmaceutics15041070 (PMC10145000; doi:10.3390/pharmaceutics15041070)
Supplement: Supplementary file 1 [file pharmaceutics-15-01070-s001.zip › pharmaceutics-2237290-supplementary.pdf]

```r
### Expiry time, process capability and release limits
### Alexis Oliva and Matías Llabrés
### Universidad de La Laguna
rm(list = ls(all = TRUE))
# libraries ---------------------------------------------------------------
require(MASS)
library(nlme)
library(lmeresampler)
require(lattice)
library(gridExtra)
library(kableExtra)
library(mgcv)
library(car)
library(cmna)
library(NLRoot)
# Users defined functions -------------------------------------------------
cpk.boot <- function(obj) {
  mu <- fixed.effects(obj)
  S <- VarCorr(obj)
  s <- sqrt(sum(as.numeric(S[, 1])))
  min((1.05*1.454 - mu)/(3*s) , (mu-1.454*0.95)/(3*s))}
cpk98.boot <- function(obj) {
  mu <- fixed.effects(obj)
  S <- VarCorr(obj)
  s <- sqrt(sum(as.numeric(S[, 1])))
  min((1.03*1.454-mu)/(3*s) , (mu-0.97*1.454)/(3*s))}

cpk <- c(1, 1.33, 1.66, 2)
r <- matrix(1 - c(2*pnorm(3*cpk)-1 , pnorm(3*cpk)),
        byrow = FALSE, ncol = 2)
r <- cbind(
```

```
  c(3, 4, 5, 6),
  format(cpk, digits = 3),
  format(r[, 1], scientific = TRUE, digits = 4),
  format(r[, 2], scientific = TRUE, digits = 4))
colnames(r) <- c("sigma level", "Cpk", "lower", "upper")

#### Linear calibration
#### Uncertainty determination by delta method
mdelta <- function(oblm, y0, f) {
  np <- length(y0)
  Z <- matrix(ncol = 3, nrow = np)
  b <- coef(oblm)
  s2r <- (sigma(oblm))^2
  S <- rbind(cbind(vcov(oblm), c(0, 0)), c(0, 0, s2r))
  for(i in 1:np) {
    Z[i,1] <- y0[i]
    Z[i,2] <- f[i] * (y0[i] - b[1])/b[2]
    yp <- c(-1/b[2] , -(y0[i] - b[1])/b[2]^2 , 1/b[2])
    Z[i,3] <- f[i] * sqrt(t(yp) %*% S %*% yp)  }
  Z <- as.data.frame(Z)
  colnames(Z) <- c("yobs", "xpred", "U")
  Z}

### Mood / Lindgren method for joint confidence for the mean and variance
### of a normal distribution
### Arnold y Shavelle (1998)
### Arguments
###    mx : sample mean
###    sx : sample standard deviation
###    n : sample siza
###    alpha : signification level
```

```
lindgren <- function(mx, sx, n, alpha = 0.05) {
  a1 <- (1-sqrt(1-alpha))/2
  a2 <- 1-sqrt(1-alpha)
  z <- qnorm(1-a1)
  chi2 <- qchisq(a2, n-1)
  lmcl <- mx - z*sx/sqrt(n)
  umcl <- mx + z*sx/sqrt(n)
  uscl <- sx * sqrt((n-1)/chi2)
  return(c(lmcl, umcl, uscl))}

aceptab1 <- function(n, xm, Linf, Lsup, Clevel = 0.95, Lbound = 0.95){
  a1 <- (1-sqrt(Clevel))/2
  a2 <- 1 - sqrt(Clevel)
  z <- qnorm(a1)
  chi2 <- qchisq(1 - sqrt(Clevel), n-1)

  f0 <- function(x) {
    s <- sqrt((n - 1)*x*x/chi2)
    Lxm <- xm - z * s/sqrt(n)
    Uxm <- xm - z * s/sqrt(n)
    p1L <- (pnorm(Lsup, Lxm, s) - pnorm(Linf, Lxm, s))^n
    p1U <- (pnorm(Lsup, Uxm, s) - pnorm(Linf, Uxm, s))^n
    OVERBD <- min(p1L, p1U)
    Lbound - OVERBD  }
  R <- bisection(f0, 0.01, 20)
  R}

rubsteg <- function(mydat, cnames, D, bt = 0.80) {
    flm <- function(xlm) {
    x <- xlm$model[, 2]
```

```
    w <- sum((x-mean(x))^2)
    c(coef(xlm), (sigma(xlm))^2, w) }

  colnames(mydat) <- cnames
  pooled.lm <- lm(y ~ batch * times, data = mydat)
  nbs <- length(levels(mydat$batch))
  batch.lm <- lapply(levels(mydat$batch),
                 function(x) lm(y ~ times, data = mydat, subset = mydat$batch == x))
  T1 <- as.data.frame(matrix(sapply(batch.lm, flm), byrow = TRUE, nrow = nbs))
  colnames(T1) <- c("b0", "b1", "s2", "w")
  b1bar <- with(T1, sum(w*b1/sum(w)))
  hav <- c(rep(0, nbs - 1), D)
  Habar <- with(T1, sum(w*hav)/sum(w))
  SSH <- with(T1, sum(w*(hav - Habar)^2))
  df1 <- length(hav) - 1
  df2 <- pooled.lm$df.residual
  s2e <- (sigma(pooled.lm))^2
  l <- SSH/s2e
  fc <- qf(1 - bt, df1, df2, l)
  ac <- 1 - pf(q = fc, df1, df2, ncp = 0)
  list(c(lambda = l, alfa.c = ac), T1)}

# EXPERIMENTAL DATA -------------------------------------------------------
# Insulin HPLC calibration data ------------------------------------------
###   name      type     description
###   conc      numeric  insuline concentration (mcg/ml)
###   day       factor   day assay
###   area      numeric  signal HPLC area
calib <- data.frame(
  conc = rep(2:8, 4),
  day = factor(rep(1:4, each = 7)),
```

```
  area = c(125784, 206639, 310492, 397939, 515037, 606082, 681455,
           129919, 211409, 313445, 403090, 502397, 599772, 678263,
           131293, 228186, 290041, 410659, 502473, 588894, 669516,
           128369, 218930, 311214, 412953, 511055, 588397, 675168)/10000)

### ----- HPLC linear calibration
calib.lm <- lm(area ~ conc * day, data = calib,
              contrasts = list(day = "contr.sum"))
calib.aov <- Anova(calib.lm, type = "III")       # H0 hypothesis batch and batch * slope
calib.lm <- update(calib.lm, area ~ conc)        # accepted and updating calibration
calib.b <- coef(calib.lm)                        # statistical model

# Process capability data --------------------------------------------
###   name     type     description
###    id      numeric    id observation
###   batch    factor     batch id
###   column   factor     HPLC column id
###   area     numeric    HPLC area signal insulin *10^(-4)
###    y       numeric    insulin concentration as % labeled content

insulin <- data.frame(
  id = 1:144,
  batch = factor(c(rep(1, 22), rep(2, 22), rep(3, 34),
                 rep(4, 28), rep(5, 30), rep(6, 8))),
  column = factor(c(rep(1, 28), rep(2, 44), rep(3, 34),
                  rep(4, 30), rep(5, 8))),
  area = c(61.6206, 62.2318, 61.9444, 61.5230, 62.5780,
           62.3175, 61.9942, 62.1160, 62.3179, 61.9941,
           62.4460, 61.5880, 61.9562, 61.3584, 62.1326,
           61.3444, 62.2910, 62.3038, 62.3530, 61.2844,
           62.4842, 62.3339, 61.5956, 61.9877, 62.3648,
```

61.6725, 61.5744, 61.6911, 62.2634, 62.4382,

61.1711, 61.7460, 61.5836, 61.9454, 61.7702,

61.7513, 62.1404, 62.3264, 62.1909, 61.8748,

61.6997, 62.4375, 62.0524, 61.6259, 62.2627,

62.4552, 61.9673, 62.2636, 61.8928, 61.5118,

61.9756, 62.4688, 62.0376, 62.2095, 61.5125,

62.1148, 61.8923, 62.2658, 62.2014, 61.7050,

62.2423, 61.8896, 61.6746, 62.2386, 62.1309,

61.8859, 61.4374, 61.9436, 61.8583, 61.4475,

62.3769, 62.4864, 61.5286, 62.2240, 60.7467,

62.6855, 62.04766, 61.9550, 61.9591, 62.5337,

61.5126, 62.3432, 62.2454, 62.1960, 62.4724,

63.1872, 62.4029, 62.3596, 61.6614, 62.1055,

61.1203, 61.6380, 61.8924, 62.4891, 62.2271,

62.6151, 61.6691, 62.2307, 62.3643, 62.6678,

62.0143, 62.3760, 61.8955, 61.4864, 61.4307,

62.3350, 62.6105, 62.6749, 62.6629, 62.2010,

62.1084, 61.6191, 61.6875, 61.9194, 61.4353,

61.5020, 61.9689, 62.2593, 62.6741, 61.7016,

62.4064, 62.0239, 62.8851, 62.2362, 62.4176,

61.5387, 62.2290, 62.7649, 62.6293, 62.2004,

62.1901, 61.8892, 62.9207, 62.2276, 62.4537,

62.6418, 62.4877, 61.7418, 61.8574, 62.0477,

61.4828, 62.6862, 62.0110, 62.6799))

Insulin stability data --

name type description

batch factor batch id

times numeric sampling time (months)

y numeric insulin concentration as % labeled content

```
tst <- c(0, 1, 3, 6, 9, 12, 18, 24)
stab <- data.frame(
  batch = factor(c(rep(1, 8), rep(2, 8), rep(3, 3), rep(4, 8),
                 rep(5, 3), rep(6, 2))),
  times = c(tst, tst, 0, 3, 6, tst, 0, 1, 3, 0, 3),
  area = c(62.2238, 60.6547, 61.1410, 59.9767, 58.9610, 56.8451, 52.8287, 50.5821,
        61.9512, 61.0012, 60.9981, 60.0448, 59.2278, 56.8462, 53.8297, 50.4458,
        61.7564, 59.7102, 59.5014,
        62.3598, 61.6787, 60.5894, 60.4532, 58.5470, 56.2327, 54.0551, 49.8325,
        61.7467, 61.4765, 61.4745,
        62.0192, 61.0187))
rm(tst)
# selected batches for stability testing
# only t <= 12 months
stab <- stab[stab$batch %in% c(1, 2, 4), ]
stab <- stab[stab$times <= 12, ]
stab <- droplevels(stab)

# STATISTICAL ANALYSIS ---------------------------------------------------
# Process capability -----------------------------------------------------
### ----- insulin concentration determination from linear calibration
### ----- and uncertainty determination by delta method

insulin <- cbind(insulin,
               mdelta(calib.lm, insulin$area, rep(0.2, length(insulin$area))))
colnames(insulin) <- c("id", "batch", "column", "area", "area2", "y", "U")
uncert <- mdelta(calib.lm, mean(insulin$area), 0.2)
insulin.A <- insulin[insulin$batch %in% c(1, 2, 4), ]
### ---------- sample statistics
### output in table 2
```

```
fcpk <- function(x) {
  L <- 0.95*1.454
  U <- 1.05*1.454
  xm <- mean(x)
  xs <- sd(x)
  cpk <- min((U-xm)/(3*xs), (xm - L)/(3*xs))
  cpk}

stat.T1 <- aggregate(insulin$y, by = list(insulin$batch),
                     function(x) c(
                       format(length(x), scientific = FALSE, digits = 0),
                       format(min(x), scientific = FALSE, digits = 4),
                       format(max(x), scientific = FALSE, digits = 4),
                       format(mean(x), scientific = FALSE, digits = 4),
                       format(sd(x), scientific = TRUE, digits = 4),
                       format(fcpk(x), scientific = FALSE, digits = 3)) )
stat.T1[[2]] <- cbind(c("A", "A", "B", "A", "B", "B"),
                 as.character(1:6),
                 stat.T1[[2]])
colnames(stat.T1[[2]]) <- c("group", "batch", "n", "min", "max", "mean", "sd", "Cpk")
stat.T1 <- as.data.frame(stat.T1[[2]])
stat.T1 <- stat.T1[order(stat.T1$group), ]

### ---------- Process capability index
### parameter estimation model in equation ___
### results in table 4
insulin.lme <- lme(y ~ 1, random = ~ 1 | batch, data = insulin.A)
insu_boot <- bootstrap(insulin.lme, .f = cpk.boot, type = "parametric", B = 1000)
insulin.boot.T2 <- confint(insu_boot)
insulin.boot.T2 <- as.data.frame(insulin.boot.T2)
```

```
insulin.lmeInt <- intervals(insulin.lme)
insulin.lme.T1 <- rbind(
  insulin.lmeInt$fixed[1,],
  insulin.lmeInt$reStruct$batch,
  insulin.lmeInt$sigma )
row.names(insulin.lme.T1) <- c("intercept", "batch sd", "residual sd")
mu.hat <- insulin.lme.T1[1,2]
s.hat <- sqrt(sum(insulin.lme.T1[2:3, 2]^2))
s.upperhat <- sqrt(sum(insulin.lme.T1[2:3, 3]^2))
cpk.hat <- min((1.05*1.454 - mu.hat)/(3*s.hat) , (mu.hat - 0.95*1.454)/(3*s.hat))
rho.hat <- insulin.lme.T1[2,2]/sum(insulin.lme.T1[2:3, 2])   # intra-class cor. coef.

# LCL <- 1.454 - 3*s.hat         # lower capability limit
# UCL <- 1.454 + 3*s.hat         # upper capability limit
LCL <- 1.462 - 3*s.hat
UCL <- 1.462 + 3*s.hat
# Stability ----------------------------------------------------------------
### ---------- Stability data analysis
### fixed effects model see equation
### results in tables
stab <- cbind(stab, mdelta(calib.lm, stab$area, rep(0.2, length(stab$area))))
stab.lm <- lm(xpred ~ batch * times, data = stab, contrasts = list(batch = "contr.sum"))
stab.aov3 <- Anova(stab.lm, type = "III")
stab.lm <- update(stab.lm, xpred ~ times)
Sres <- sigma(stab.lm)
### --------------- ANOVA type III
### output in table 5
stabG1 <- stab[stab$batch %in% c(1,2,4), ]
stabG1$batch <- droplevels(stabG1$batch)
stabG1.lm <- lm(xpred ~ times*batch, data = stabG1)
s2e <- sigma(stabG1.lm)^2
```

```
stab.aov3 <- Anova(stabG1.lm, type = "III")
stab.aov3.T5 <- cbind(
  row.names(stab.aov3),
  format(stab.aov3[[1]], scientific = TRUE, digits = 4),
  format(stab.aov3[[2]], scientific = FALSE, digits = 1),
  format(stab.aov3[[1]]/stab.aov3[[2]], scientific = TRUE, digits = 4),
  format(stab.aov3[[3]], scientific = FALSE, digits = 3),
  format(round(stab.aov3[[4]], 3), scientific = FALSE, digits = 3))
colnames(stab.aov3.T5) <- c("Source Variation", "SSQ", "DF", "MS", "F", "Pr(>F)")

### ---------- Method of Ruberg and Stegeman (1991)
flm <- function(xlm) {
  x <- xlm$model[, 2]
  w <- sum((x-mean(x))^2)
  c(coef(xlm), (sigma(xlm))^2, w)}

E1 <- lapply(as.factor(c(1,2,4)),
          function(x) lm(xpred ~ times, data = stabG1, subset = stabG1$batch == x))
T1 <- as.data.frame(matrix(sapply(E1, flm), byrow = TRUE, nrow = 3))
colnames(T1) <- c("b0", "b1", "MSE", "w")

rubsteg.out <- rubsteg(stab, c("batch", "times", "area", "yobs", "y", "U"),
          -(0.02*0.95*1.454)/4.10, 0.90)
Delta <- -(0.02*0.95*1.454)/4.10
tshelf <- numeric(3)
for(i in 1:3) {
  prd <- predict.lm(E1[[i]], data.frame(times = 0:12),
              level = 0.90, interval = "confidence")[, 2]
  funprd <- splinefun(prd, 0:12)
  tshelf[i] <- funprd(0.95*1.454)}
```

```
T1.out <- cbind(
  format(c("1", "2", "3", "pooled"), scientific = FALSE, digits = 1),
  format(c(T1[, 1], NA), scientific = FALSE, digits = 4),
  format(c(T1[, 2], NA), scientific = TRUE, digits = 4),
  format(c(sqrt(T1[, 3]), NA), scientific = TRUE, digits = 4),
  format(c(tshelf, NA), scientific = FALSE, digits = 3))
colnames(T1.out) <- c("batch", "b0", "b1", "RSD", "T[ICH]")

### ---------- model prediction and shelf-life estimation
stab.pred <- cbind(stab,
            predict(stab.lm, stab, interval = "confidence",
                 level = 0.90)  )
z <- predict.lm(stab.lm, data.frame(times = stab$times),
             interval = "prediction", level = 0.90)[, 2]
stab.pred <- cbind(stab.pred, z)

stab.fun1 <- with(stab.pred[stab.pred$batch == 1, ], splinefun(lwr, times))
T0 <- stab.fun1(0.95*1.454)
stab.fun2 <- with(stab.pred[stab.pred$batch == 1, ], splinefun(z, times))
T1 <- stab.fun2(0.95*1.454)

### actualization T1.out table
### see table 4 - manuscript
T1.out[4,2] <- format(coef(stab.lm)[1], scientific = FALSE, digits = 4)
T1.out[4,3] <- format(coef(stab.lm)[2], scientific = TRUE, digits = 4)
T1.out[4,4] <- format(sigma(stab.lm), scientific = TRUE, digits = 4)
T1.out[4,5] <- format(T0, scientific = FALSE, digits = 3)

# Release limits -------------------------------------------------------
### Method of Chen & van der Vaart (2022)
#
```

```
fadg <- function(x) {
  s2b <- vcov(stab.lm)[2,2]
  b <- coef(stab.lm)[2]
  s <- s.hat
  LCL - 1.454*0.95 + b*x - 2*sqrt(x^2*s2b + s^2)}

T0.adg <- bisection(fadg, 0, 8)
c(T0, T0.adg)
y0.adg <- 0.95*1.454 - coef(stab.lm)[2]*T0.adg + 2*sqrt(T0.adg^2*vcov(stab.lm)[2,2]+s.hat^2)
c(LCL, y0.adg)

fadg2 <- function(x) {
  s2b <- vcov(stab.lm)[2,2]
  b <- coef(stab.lm)[2]
  s <- s.hat
  1.454*0.95 - b*x + 2*sqrt(x^2*s2b + s^2)}

# ASTM ------------------------------------------------------------------
ft <- function(x, xm, nsamp) {
  n <- nsamp; Linf <- LCL; Lsup <- UCL
  Clevel <- 0.90; Lbound <- 0.95
  a1 <- (1-sqrt(Clevel))/2
  a2 <- 1 - sqrt(Clevel)
  z <- qnorm(a1)
  chi2 <- qchisq(1 - sqrt(Clevel), n-1)
  s <- sqrt((n - 1)*x*x/chi2)
  Lxm <- xm - z * s/sqrt(n)
  Uxm <- xm + z * s/sqrt(n)
  p1L <- (pnorm(Lsup, Lxm, s) - pnorm(Linf, Lxm, s))^n
  p1U <- (pnorm(Lsup, Uxm, s) - pnorm(Linf, Uxm, s))^n
```

```
  OVERBD <- min(p1L, p1U)
  1 - OVERBD}
###
astm.T1 <- data.frame(
  pc = seq(0.985, 1.015, length = 101),
  xm = 1.462*seq(0.985, 1.015, length = 101),
  sl10 = numeric(101),
  p10 = numeric(101),
  n10 = numeric(101),
  sl30 = numeric(101),
  p30 = numeric(101),
  n30 = numeric(101))

for(i in 1:101) {
x <- 1e-3
xm <- astm.T1$xm[i]
n <- 0
# c(n, 1.454*pr, x, ft(x, pr))

while(ft(x, xm, 10) <=  0.95 & n < 1500) {
  x <- x + 1e-5
  n <- n+1}
astm.T1$sl10[i] <- x
astm.T1$p10[i] <- ft(x, xm, 10)
astm.T1$n10[i] <- n}

plot(astm.T1$xm, astm.T1$sl10, type = "l")
points(stat.T1$mean[1:3], stat.T1$sd[1:3])
points(stat.T1$mean[4:6], stat.T1$sd[4:6], pch = 12)
abline(v = c(1.454, 1.462), lty = c(2,1))
```

```
for(i in 1:101) {
  x <- 1e-3
  xm <- astm.T1$xm[i]
  n <- 0
  # c(n, 1.454*pr, x, ft(x, pr))
  while(ft(x, xm, 30) <=  0.95 & n < 1500) {
    x <- x + 1e-5
    n <- n+1  }
  astm.T1$sl30[i] <- x
  astm.T1$p30[i] <- ft(x, xm, 30)
  astm.T1$n30[i] <- n}

plot(astm.T1$xm, astm.T1$sl10, type = "l", ylim = c(0, 0.012),
     xlab = "mean", ylab = "standard deviation")
lines(astm.T1$xm, astm.T1$sl30)
points(stat.T1$mean[1:3], stat.T1$sd[1:3], pch = 1)
points(stat.T1$mean[4:6], stat.T1$sd[4:6], pch = 2)
astm.T2 <- astm.T1[, c(2,3,6)]
### Montes

# stab.new <- stab
# colnames(stab.new) <- c("Lot", "Month", "area1", "area2", "Y", "U")
# montes <- WithSlope(stab.new, T0.adg, 0.95, 0.95)
# montes
# c(T, LRL, cot.irl)
# montes
### Figures
# insu_boot98 <- bootstrap(insulin.lme, .f = cpk98.boot, type = "parametric", B = 1000)
# insulin.boot98.T2 <- confint(insu_boot98)
# insulin.boot98.T2 <- as.data.frame(insulin.boot98.T2)
```

```
# FIGURES ----------------------------------------------------------------
jpeg("figure_0.jpeg", width = 480, height = 680, quality = 100)
par(mfrow = c(1,2))
plot(area ~ conc, data = calib[calib$day == 1, ], type = "b", lty = 2,
    xlim = c(0, 10), ylim = c(0, 75),
    ylab = list("HPLC signal area", cex = 1.25),
    xlab = list(expression(paste("insulin concentration (", mu,"g/ml)")), cex = 1.25))
for(i in 2:4) lines(area ~ conc, data = calib[calib$day == i,],
                type = "b", lty = 2)
abline(calib.lm)

calib <- within(calib, ratio <- (area - coef(calib.lm)[1])/conc)
plot(ratio ~ conc, data = calib[calib$day == 1, ], type = "b", lty = 2,
    xlim = c(0, 10), ylim = c(8.5, 10),
    xlab = list(expression(paste("insulin concentration (", mu,"g/ml)")), cex = 1.25))
for(i in 2:4) lines(ratio ~ conc, data = calib[calib$day == i, ],
                type = "b", lty = 2)
abline(h = c(coef(calib.lm)[2], confint(calib.lm)[2,]), lty = 2)
dev.off()

jpeg("figure_1.jpeg", width = 680, height = 480, quality = 100)
par(mfrow = c(1,2))
fig_1 <- plot(y ~ batch, data = insulin,
            ylim = c(1.35, 1.55),
    ylab = list("Insulin concentration", cex = 1.25),
    xlab = "Batch",
    cex = 1.25)
abline(h = c(1.454*c(0.95 , 1, 1.05), 1.462), lty = 2)
abline(h = c(UCL, LCL), lty = 2)
text(1.5, 1.39, "LSL", cex = 1.25)
```

```
text(1.5, 1.535, "USL", cex = 1.25)
text(1.5, 1.431, "LCL", cex = 1.25)
text(1.5, 1.494, "UCL", cex = 1.25)
fig_2 <- qqnorm(insulin$y,
      xlim = c(-3, 3), ylim = 1.454*c(0.975, 1.025))
qqline(insulin$y)
dev.off()

##### ----- Figure 2
jpeg("figure_2.jpeg", width = 680, height = 680, quality = 100)
plot(xpred ~ times, data = stab[stab$batch == 1,], type = "b", lty = 2,
    ylim =c(1.30, 1.50), xlim = c(0, 15),
    cex.axis = 1.5,
    xlab = list("Time (months)", cex = 1.5),
    ylab = list("Insuline concentration", cex = 1.5))
for(i in c(2,4)) points(xpred ~ times, data = stab[stab$batch == i, ],
                  type = "b", lty = 2, pch = i)
abline(h = c(0.95, 1, 1.05)*1.454, lty = 2)     # LSL & USL
abline(h = c(UCL, LCL), lty = 2)                # 3*sigma capability limits
lines(stab.pred[stab.pred$batch == 1, c(2,7)])                  # fit
lines(stab.pred[stab.pred$batch == 1, c(2,8)], lty = 2, lwd = 2)   # confidence
lines(stab.pred[stab.pred$batch == 1, c(2, 10)], lty = 4, lwd = 2) # prediction
# abline(a = y0.adg, b = coef(stab.lm)[2])
segments(T0, 0, T0, 0.95*1.454, lty = 2)
segments(T1, 0, T1, 0.95*1.454, lty = 2)
text(12.5, UCL+0.005, "UCL", cex = 1.5)
text(12.5, LCL+0.005, "LCL", cex = 1.5)
text(12.5, 1.454+0.005, "TARGET", cex = 1.5)
text(12.5, 0.95*1.454+0.005, "LSL", cex = 1.5)
text(9.5, 1.305, expression(T[ICH]), cex = 1.5)
text(10.5, 1.305, " = ", cex = 1.5)
```

```
text(12, 1.305, paste(round(T0, 1)," months"), cex = 1.5)
text(4.5, 1.31, expression(T[pred]), cex = 1.5)
text(5.5, 1.31, " = ", cex = 1.5)
text(7, 1.31, paste(round(T1, 2)," months"), cex = 1.5)
dev.off()

### --------------- figure 3
jpeg("figure_3.jpeg", width = 680, height = 680, quality = 100)
curve(fadg2, from = 1, to = 8,
      cex = 1.5, cex.axis = 1.5,
      ylim = c(1.4, 1.5),
      xlab = list("Shelf life (months)", cex = 1.5),
      ylab = list(expression(LRL[ADG]), cex = 1.5))
abline(h = c(LCL, 1.454, 1.462, UCL), lty = 2)
text(7, LCL+0.002, "LCL / LRL", cex = 1.5)
text(3, 1.454+0.002, "LABELED CONCENTRATION", cex = 1.5)
text(3, 1.462 + 0.002, "MEAN PROCESS", cex = 1.5)
text(7, UCL+0.002, "UCL / URL", cex = 1.5)
segments(T0.adg, 1, T0.adg, LCL, lty = 2)
text(3.5, 1.405, expression(T[ADG]), cex = 1.5)
text(4.0, 1.405, " = ", cex = 1.5)
text(4.8, 1.405, paste(round(T0.adg, 2), " months"), cex = 1.5)
dev.off()

jpeg("figure_4.jpeg", width = 680, height = 680, quality = 100)
plot(astm.T1$xm, astm.T1$sl10, type = "l", ylim = c(0, 0.012),
     cex = 1.5, cex.axis = 1.5,
     xlab = list("Mean concentration", cex = 1.5),
     ylab = list("Standard deviation", cex = 1.5))
lines(astm.T1$xm, astm.T1$sl30)
points(stat.T1$mean[1:3], stat.T1$sd[1:3], pch = 1, cex = 2)
```

```
points(stat.T1$mean[4:6], stat.T1$sd[4:6], pch = 2, cex = 2)

dev.off()
```
